# Unraveling the Role of JMJD1B in Genome Stability and the Malignancy of Melanomas

**DOI:** 10.3390/ijms251910689

**Published:** 2024-10-04

**Authors:** Perla Cruz, Diego Peña-Lopez, Diego Figueroa, Isidora Riobó, Vincenzo Benedetti, Francisco Saavedra, Claudia Espinoza-Arratia, Thelma M. Escobar, Alvaro Lladser, Alejandra Loyola

**Affiliations:** 1 Centro Ciencia & Vida, Fundación Ciencia & Vida, Santiago 8580702, Chiledfigueroa@cienciavida.org (D.F.); alladser@cienciavida.org (A.L.); 2 Department of Biochemistry, University of Washington, Seattle, WA 98195, USA; 3 Institute for Stem Cell and Regenerative Medicine, University of Washington, Seattle, WA 98195, USA; 4 Facultad de Medicina y Ciencia, Universidad San Sebastián, Santiago 7510602, Chile

**Keywords:** melanoma, chromatin structure, DNA damage, histone protein levels, genome instability, JMJD1B

## Abstract

Genome instability relies on preserving the chromatin structure, with any histone imbalances threating DNA integrity. Histone synthesis occurs in the cytoplasm, followed by a maturation process before their nuclear translocation. This maturation involves protein folding and the establishment of post-translational modifications. Disruptions in this pathway hinder chromatin assembly and contribute to genome instability. JMJD1B, a histone demethylase, not only regulates gene expression but also ensures a proper supply of histones H3 and H4 for the chromatin assembly. Reduced JMJD1B levels lead to the cytoplasmic accumulation of histones, causing defects in the chromatin assembly and resulting in DNA damage. To investigate the role of JMJD1B in regulating genome stability and the malignancy of melanoma tumors, we used a JMJD1B/KDM3B knockout in B16F10 mouse melanoma cells to perform tumorigenic and genome instability assays. Additionally, we analyzed the transcriptomic data of human cutaneous melanoma tumors. Our results show the enhanced tumorigenic properties of JMJD1B knockout melanoma cells both in vitro and in vivo. The γH2AX staining, Micrococcal Nuclease sensitivity, and comet assays demonstrated increased DNA damage and genome instability. The JMJD1B expression in human melanoma tumors correlates with a lower mutational burden and fewer oncogenic driver mutations. Our findings highlight JMJD1B’s role in maintaining genome integrity by ensuring a proper histone supply to the nucleus, expanding its function beyond gene expression regulation. JMJD1B emerges as a crucial player in preserving genome stability and the development of melanoma, with a potential role as a safeguard against oncogenic mutations.

## 1. Introduction

Genome instability, one of the hallmarks of cancer, is broadly associated with mutations in the DNA repair machinery and/or mitotic checkpoint genes [1,2]. However, the chromatin structure also impacts genome stability [3]. An excess and restraint of the histone protein levels are toxic to the cell and can lead to DNA damage and genome instability [4,5,6,7,8,9]. Therefore, histone dynamics is a tightly regulated process, wherein synthesis occurs during the S phase when DNA is replicated, followed by the reassembly into chromatin [10,11]. Histone synthesis takes place in the cytoplasm by free ribosomes, after which they are translocated to the nucleus. However, before nuclear translocation, newly synthesized histones H3 and H4 undergo a maturation process where they acquire the correct protein folding and post-translational modifications [12,13,14]. In this pathway, histones H3/H4 interact with different protein chaperones, enzymes, and histone-associated proteins. These include chaperones like Hsp90, Hsp70, and Hsc70; enzymes such as HAT1; and histone chaperones like sNASP (somatic NASP), tNASP (testis NASP), ASF1, and RbAp48; as well as histone-associated proteins like PP32/SET and Importin 4. While histone H3 is being translated, it undergoes its first modification, in which the monomethylation of lysine 9 of histone H3 is mediated by SetDB1, which associates with ribosomes. Following their synthesis, newly formed histones H3 and H4 first interact with heat shock proteins and PP32/SET proteins, which regulate the acetylation levels of histone H4. The next step in the maturation cascade is the dimerization of H3 and H4 via their histone fold domains, a reaction facilitated by the chaperones tNASP and HSP90, which serves as a quality control checkpoint. After the dimerization, the H3-H4 dimers associate with sNASP, ASF1, and HAT1, where H4 undergoes acetylation by HAT1. Finally, the H3-H4 dimers interact with Importin 4 and ASF1 to facilitate their translocation into the nucleus through the nuclear pore [4,12,13,15,16]. Importantly, alterations in any component of the histone/chaperone network, whether during histone maturation or chromatin assembly, can impact chromatin integrity and cell cycle progression, leading to DNA damage and genome instability [4,15,16,17,18,19].

JMJD1B, also known as JHDM2B, which is encoded by the KDM3B gene, is a histone demethylase member of the JMJD1 family of enzymes with specificity for mono- and dimethylated lysine 9 of histone H3 (H3K9) [20,21] and dimethylated arginine 3 of histone H4 (H4R3) [22]. The human KDM3B gene, encoding the JMJD1B protein, is in the 5q31 locus of the genome. Deletions in the human chromosome 5 (del(5q)) are commonly observed in malignant myeloid disorders. Due to its location in this locus, JMJD1B was initially identified as a candidate tumor suppressor gene [23,24]. The JMJD1 family also includes JMJD1A and JMJD1C. JMJD1A and JMJD1B share 59.6% of their amino acid sequences, while neither of them shares more than 50% of its sequence with JMJD1C. This suggests that JMJD1C is evolutionarily distinct [25]. JMJD1A and JMJD1B usually activate the transcription of their target genes due to the demethylation of the repressive H3K9 methylation mark [26,27]. JMJD1B knockout mice are viable but infertile and have cell growth and hematopoietic development defects [22,28]. In contrast, JMJD1A/JMJD1B double knockout mice are embryonically lethal soon after implantation [29], indicating redundancy between the two proteins. The double knockout correlates with the increased levels of H3K9me2/3 in the euchromatic regions of the genome, suggesting a role for the JMJD1A/JMJD1B proteins in establishing an appropriate H3K9 methylated state in early embryogenesis [29].

Interestingly, the JMJD1B protein functions beyond the regulation of gene expression. In a previous study, we demonstrated that JMJD1B ensures the appropriate supply of histones H3 and H4, thereby preserving genome integrity [4]. A reduction in the JMJD1B protein levels in the HeLa cells leads to the misregulation of the histone chaperone tNASP proteolysis, resulting in the accumulation of tNASP and newly synthesized histones H3 and H4 in the cytoplasm. This cytoplasmic accumulation of tNASP and histones affects the supply of histones H3 and H4 to the nucleus, causing defects in chromatin assembly, γH2AX staining, and G2/M cell cycle arrest—all are indicative of DNA damage. Furthermore, through the analysis of the publicly available datasets from patients affected by various types of cancer, we established a positive correlation between JMJD1B gene mutations, cancer incidence, and genome instability [4]. However, we did not investigate how a reduction in the JMJD1B protein levels would affect genome stability. Given that previous studies suggested that JMJD1B acts as a tumor suppressor [22,23,30], we aimed to directly investigate whether JMJD1B ensures genome stability and whether its loss leads to chromosome instability. To achieve this, we knocked out the JMJD1B/KDM3B gene in the B16F10 mouse melanoma cell line. Our findings indicate an enhancement in all the tumorigenic properties investigated upon the JMJD1B knockout. Moreover, γH2AX staining, Micrococcal Nuclease sensitivity, and comet assays revealed an increased genome instability in JMJD1B knockout B16F10 cells compared with wild-type cells. An analysis of the human melanoma tumors databases reveals that JMJD1B expression correlates with a lower overall mutational burden and oncogenic driver mutations. Hence, we propose that the JMJD1B protein contributes to maintaining genome integrity by ensuring the histone supply to the nucleus, extending beyond its role in gene expression.

## 2. Results

### 2.1. Histones H3 and H4 and NASP Accumulate in Cytosolic Extracts upon JMJD1B Depletion

**Figure 1:**
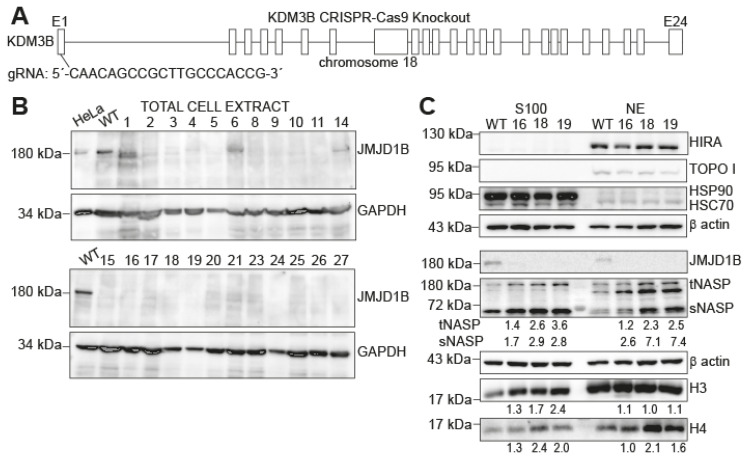
**Accumulation of histones H3 and H4 and NASP in cytosolic extracts upon JMJD1B depletion.** (**A**) Scheme of the mouse JMJD1B/KDM3B knockout using the CRISPR-Cas9 technique. (**B**) Western blot analysis of 5 µg of total cell extract from the wild type (WT) and clones obtained from the JMJD1B gene knockout using the CRISPR/Cas9 technique. The total cell extract from the HeLa cells served as a control for the JMJD1B signal. From these clones, we selected three (clones #16, #18, and #19), based on their growth characteristics, phenotypic traits, and lack of JMJD1B expression, as determined by Western blot analyses, for further investigation. Clones #7, #12, #13, #17, and #22 were excluded from the analysis due to poor growth or abnormal cell morphology. (**C**) Western blot analysis of 5 µg of cytosolic (S100) and nuclear (NE) extracts from the wild type (WT) and clones derived from the B16F10 JMJD1B KO cells, blotted as indicated. The number below the blots represent the fold changes relative to the wild type. The bands were quantified, normalized to β-actin, and then expressed as fold changes compared to the wild type. Of note is that the band below tNASP is only observed in the nuclear extract derived from mouse cells.

To directly investigate whether JMJD1B loss leads to chromosome instability, we examined the consequences of depleting the JMJD1B protein in cancer cells. We chose the B16F10 mouse melanoma cell line as a working model because of its well-established use in cancer research, its aggressive metastatic behavior, and its relevance in studying melanoma biology and tumorigenesis. Specifically, we utilized CRISPR/Cas9-mediated genome editing technology to knock out the JMJD1B/KDM3B gene in the B16F10 mouse melanoma cell line (Figure 1A). We confirmed the absence of the JMJD1B protein in several clones through a Western blot analysis and selected three clones (clones #16, #18, and #19) to continue the studies (Figure 1B). In previous work, we demonstrated that reducing the JMJD1B protein levels in HeLa cells resulted in the accumulation of histones H3 and H4, along with the chaperones NASP and HSP90, in cytosolic extracts [4]. Therefore, we obtained cytosolic (S100) and nuclear (NE) extracts from the B16F10 cells (Figure 1C). To assess the quality of the extract preparation, we analyzed HIRA and TOPO I as the nuclear markers, confirming that the cytosolic extracts were free of nuclear contamination. We also analyzed HSP90 as a cytosolic marker, confirming the enrichment of this protein in the cytosolic extract and its absence in the nuclear extract. Additionally, we confirmed the absence of the JMJD1B protein in these extracts. Subsequently, we investigated the protein levels of H3 and H4 and their associated partners in the B16F10 JMJD1B knockout cells (Figure 1C). We observed the accumulation of NASP and histones H3 and H4 in the cytosolic extracts of the JMJD1B knockout cells, compared to wild-type cells. Interestingly, histone H3 remained unchanged in the nuclear extracts. In summary, depleting the JMJD1B gene in B16F10 cells leads to the cytosolic accumulation of H3, H4, and NASP, mirroring our previous observations in HeLa cells.

### 2.2. The Tumorigenic Properties of B16F10 Cells Are Potentiated by JMJD1B Depletion

**Figure 2:**
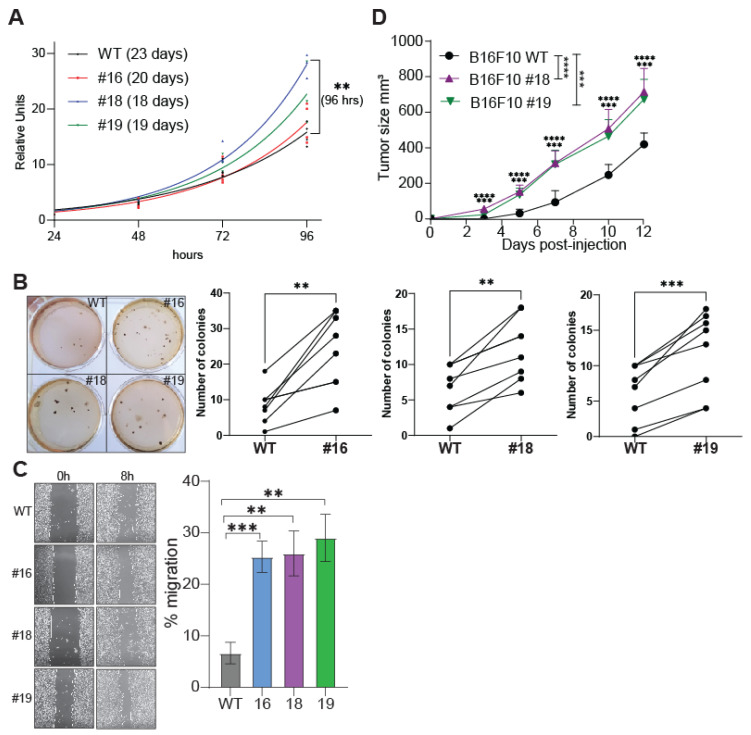
**The tumorigenic properties of B16F10 cells are potentiated upon JMJD1B depletion.** (**A**) Proliferation of B16F10 wild-type (WT) and JMJD1B knockout cells measured with the CCK-8 assay in three–four independent experiments. The doubling time for each B16F10 cell is shown in parentheses, calculated with a non-linear fit, giving R squared values of 0.94 (WT), 0.92 (#16), 0.98 (#18), and 0.92 (#19). The significance was determined by a two-way ANOVA. **, *p* value of <0.01. (**B**) Soft agar plaque assay. Representative images of the soft agar plaques and graphs displaying pairwise comparisons of the total number of colonies obtained from experiments using B16F10 wild-type (WT) cells and JMJD1B knockout clones #16, #18, and #19 are shown. Images were captured after 17 days and seven to ten biological replicates were used. The significance was determined by a paired *t*-test. **, *p* value of <0.01; and ***, *p* value of <0.001. (**C**) Wound healing assay. Representative image of three independent assays and graph displaying cell migration expressed as the percentage of migration. Wild-type and JMJD1B knockout B16F10 cells were plated on plastic dishes, wounded with a P200 pipette tip, and photographed at 0 and 8 h. The significance was determined by a *t*-test. **, *p* value of <0.01; and ***, *p* value of <0.001. (**D**) In vivo tumor growth. RAG1 KO C5BL/6 mice were intradermally injected with 1 × 10^6^ wild-type (WT) and JMJD1B knockout clones #18 and #19 B16F10 melanoma cell lines. Tumor growth was evaluated in the subsequent days. The graph presents the average tumor growth for each group, showing the mean ± SEM of the tumor sizes from one experiment, with n = 5 mice per group. The significance was determined by a two-way ANOVA and Bonferroni’s post hoc test. ***, *p* value ≤ 0.001; and ****, *p* value ≤ 0.0001.

We previously demonstrated that a reduction in JMJD1B protein levels in HeLa cells results in histone deposition defects, G2/M cell cycle arrest, and DNA damage. Furthermore, we established a positive correlation between JMJD1B gene mutations and cancer incidence and genomic instability [4]. Based on these findings, we sought to investigate the impact of depleting the JMJD1B gene on the tumorigenic properties of B16F10 cell lines. We first investigated cell proliferation and observed a modest enhancement in the proliferation of one of the JMJD1B knockout cells relative to their wild-type counterparts (Figure 2A). We then evaluated the cells’ ability to grow independently of anchorage by conducting a soft agar assay. Interestingly, all three JMJD1B knockout cells demonstrated a significantly higher number of colonies compared to the wild-type cells (Figure 2B), suggesting an augmented capacity for transformation in the absence of JMJD1B. To further explore cell migration and cell–cell interaction properties, we conducted a wound healing assay. Notably, JMJD1B knockout cells exhibited enhanced cell migration (Figure 2C). Subsequently, we investigated the tumorigenic capacities of these cells in vivo. To do so, we intradermally injected 1 × 10^6^ wild-type or JMJD1B knockout B16F10 melanoma cells into RAG1 KO mice, and the tumor growth was evaluated in the subsequent days. Remarkably, we observed that the tumors derived from the B16F10 JMJD1B knockout cells grew faster compared to those derived from the wild-type B16F10 cells (Supplementary Figure S1 and Figure 2D). Taken together, the data demonstrated that the elimination of the JMJD1B gene potentiates the tumorigenic properties of the B16F10 cells both in vitro and in vivo, suggesting that JMJD1B plays a regulatory role in cancer development.

### 2.3. Depletion of JMJD1B Increases Genomic Instability in B16F10 Cells

**Figure 3:**
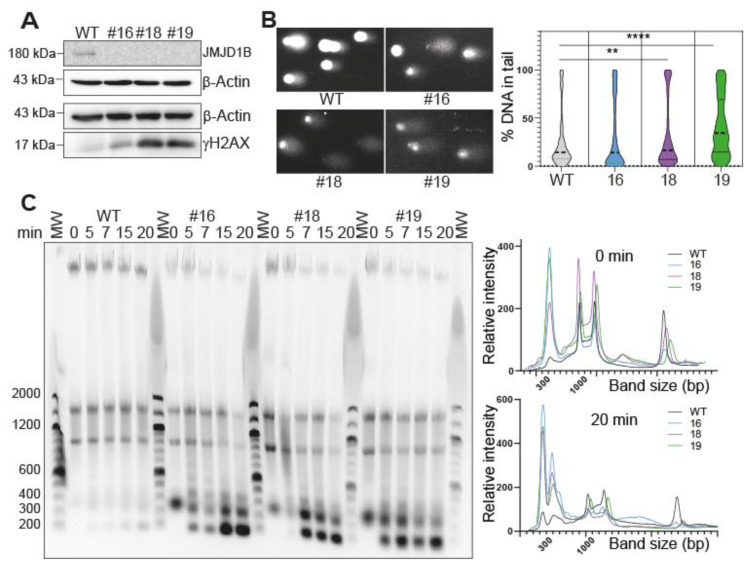
**Depletion of JMJD1B increases genomic instability in B16F10 cells.** (**A**) Western blot analysis. Western blot analysis of 20 µg of total cell extract derived from wild-type (WT) and JMJD1B knockout clones #16, #18, and #19, blotted as described. (**B**) Comet assay. Comet assay of B16F10 cells, wild-type (WT) cells, and JMJD1B knockout clones #16, #18, and #19. On the left, there is a representative stained DNA image after electrophoresis of the lysed cells. On the right, a violin graph represents the quantification of DNA found in the tail of the comets. For this assay, between 500 and 1500 cells were quantified per condition from three independent experiments. The data were analyzed with a two-way ANOVA test. **, *p* value of <0.01; and ****, *p* value of <0.0001. (**C**) Micrococcal Nuclease (MNase) digestion. MNase digestion of wild-type (WT) B16F10 cells and JMJD1B knockout clones #16, #18, and #19 was performed for the indicated times. The DNA extracted from the digested cells was analyzed on 1.5% agarose gels and visualized with a GelRed stain and UV illumination. Molecular weight (MW) markers were run between the clones, and their corresponding sizes are indicated on the left side of the gel. The graphs represent densitometric scans of the electrophoretic patterns of the MNase-digested DNA at 0 and 20 min from all the cells. The image is a representative experiment of three independent assays.

Our previous work revealed that reducing the JMJD1B protein levels leads to chromatin assembly defects and DNA damage, suggesting increased genomic instability in these cells. Additionally, we established a correlation between JMJD1B gene mutations and cancer incidence and genome instability using the NIH Cancer Data Portal [4]. Consistent with these findings, we observed here that the tumorigenic properties of the B16F10 cells are enhanced when the JMJD1B gene is knocked out, both in cultured cells and a murine model (Figure 2). Consequently, we aimed to directly investigate whether these effects could be attributed to the role of JMJD1B in maintaining genome stability. To assess genome integrity, we performed several assays that evaluated different aspects of DNA damage and the chromatin structure. First, we examined the phosphorylation of histone H2AX (γH2AX), a well-established marker for DNA damage that localizes to the sites of double-strand breaks in the genome [31,32]. A Western blot analysis using the total cell extracts from the B16F10 wild-type and JMJD1B knockout cells confirmed the absence of JMJD1B in the knockout cells (Figure 3A). Additionally, the analysis revealed an elevated γH2AX signal in the JMJD1B knockout cells compared to the wild-type cells, indicating increased DNA damage (Figure 3A). To further validate these findings, we conducted a comet assay (Figure 3B). The B16F10 cells embedded in agarose on microscope slides were subjected to lysis and electrophoresis using an alkaline buffer. We observed no difference between the wild-type B16F10 cells and JMJD1B knockout B16F10 melanoma clone #16. However, a higher number of comets was detected in the JMJD1B knockout clones #18 and #19 compared to the wild-type B16F10 cells, indicating greater DNA damage in those knockout cells. We also conducted a Micrococcal Nuclease (MNase) DNA digestion assay using nuclei derived from each of the B16F10 cell types. The MNase digestion was performed at various time points ranging from 0 to 20 min, and the extent of DNA digestion was evaluated through gel electrophoresis on an agarose gel (Figure 3C). We found that DNA from the JMJD1B knockout cells underwent faster and more extensive digestion compared to that from the wild-type cells. This observation suggests that JMJD1B knockout cells are more susceptible to MNase-mediated DNA digestion, indicating increased chromatin accessibility. Overall, these results highlight the critical role of JMJD1B in maintaining genomic stability, and its depletion leads to increased DNA damage and an altered chromatin structure in B16F10 cells.

### 2.4. JMJD1B in Human Melanoma Tumors

**Figure 4:**
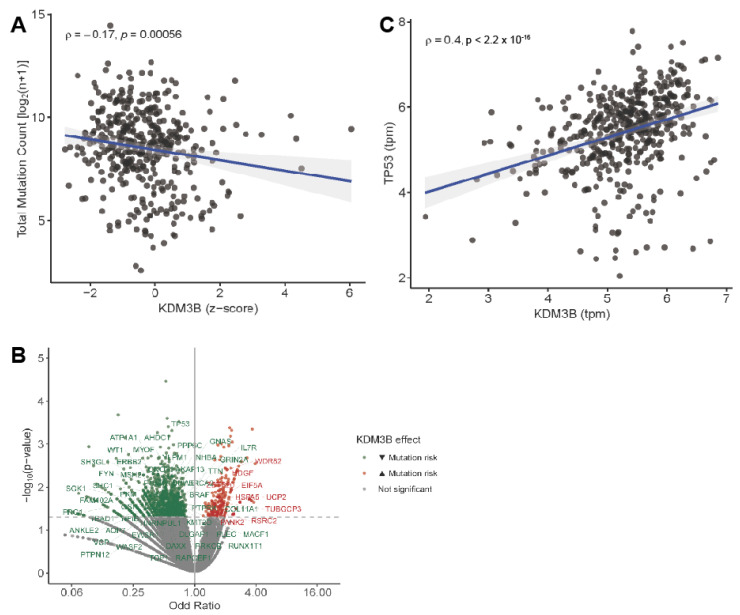
KDM3B/JMJD1B expression associates with reduced mutational burden in the genome and oncogenic drivers in human melanoma tumors. (**A**) Spearman’s rank correlation between KDM3B expression and overall mutational load in human melanoma tumors. (**B**) Volcano plot for the odds ratio and *p*-value analyzed by logistic regression showing the expression of KDM3B and the probability of SNV mutations for a majority set of tumor suppressor genes and proto-oncogenes. Genes associated with lower and higher mutation risks are identified in the upper left (in green) and upper right (in red) quadrants, respectively. (**C**) Spearman’s rank correlation between KDM3B and TP53 expression.

Given that our results demonstrate signs of chromosome instability in the JMJD1B knockout cells, we sought to investigate whether JMJD1B-mediated DNA stability plays a role in safeguarding against overall and oncogenic driver mutations in human melanoma. To address this, we examined the human melanoma (SKCM) patient database, focusing on melanoma patients. Our analysis revealed a negative correlation between JMJD1B/KDM3B expression and overall mutational burden (Figure 4A), supporting its protective role in maintaining DNA integrity. Interestingly, when we analyzed the probability of mutations in the genes associated with malignant transformation, such as tumor suppressors or oncogenes, we observed that KDM3B expression was associated with a reduced mutation risk in the tumor suppressor genes, such as TP53 and BRCA2, and proto-oncogenes, such as BRAF (Figure 4B). Indeed, JMJD1B/KDM3B expression was strongly correlated with the levels of TP53 (Figure 4C). These findings suggest that JMJD1B acts as a protective factor for the genome, safeguarding against the emergence of oncogenic driver mutations. Taken together, our findings suggest that JMJD1B plays an important role in maintaining genome stability, potentially acting as a safeguard against oncogenic mutations.

## 3. Discussion

Genome instability is a hallmark of cancer progression and is often associated with DNA mutations. However, maintaining a proper chromatin structure and the precise regulation of histone protein levels is also crucial for preserving genome integrity. In our previous studies, we demonstrated that the JMJD1B protein regulates the supply of histones from the cytoplasm to the nucleus. Decreased levels of JMJD1B result in defects in chromatin assembly, increased γH2AX staining, and G2/M cell cycle arrest, all indicative of DNA damage due to the disruption of histone delivery to the nucleus [4]. In this present study, we investigated whether JMJD1B loss leads to chromosome instability. We demonstrate that knocking out the JMJD1B/KDM3B gene in the B16F10 melanoma cell line enhances the tumorigenic properties of the cells both in vitro and in vivo and results in increased genome instability. While the H3K9 demethylation mediated by JMJD1B has been recognized for its impact on gene expression and cancer development [21,22,29,30,33,34,35], our results demonstrate that the absence of JMD1B also contributes to genome instability through the deregulation of histone distribution and delivery to the chromatin. This effect contradicts expectations associated with the depletion of an H3K9 demethylase, as it does not increase chromatin compaction. Therefore, our results highlight a new role for JMJD1B in the histone supply to chromatin and genome stability.

**Figure 5:**
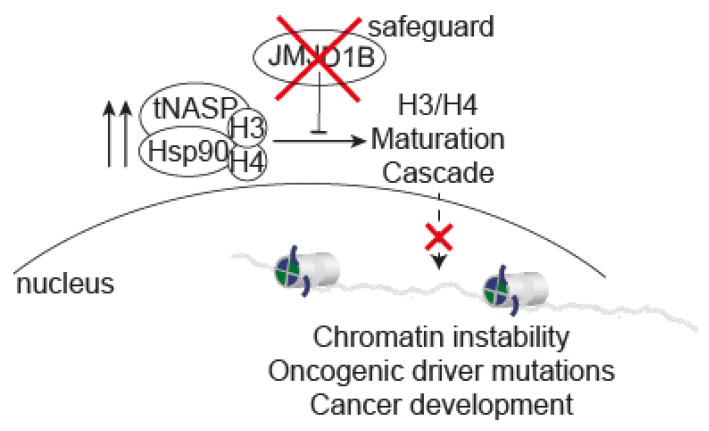
**Model of the role of JMJD1B as a regulator of chromosome stability.** Through a yet-unknown mechanism, the absence of JMJD1B leads to an increase in the protein levels of the histone chaperone tNASP, which accumulates newly synthesized histones H3/H4 in the cytoplasm, affecting the histone supply to the nucleus and leading to genome instability. This process highlights the potential role of JMJD1B in cancer progression beyond its established function in gene expression regulation: genome instability that correlates with oncogenic driver mutations, including p53.

The human KDM3B gene, encoding the JMJD1B protein, is located in the 5q31 locus of the genome. Deletions involving the human chromosome 5 (del(5q)) are commonly observed in malignant myeloid disorders, such as myelodysplasia and acute myeloid leukemia. Due to its location in this locus, JMJD1B was initially identified as a candidate tumor suppressor gene [23,24]. In fact, the re-expression of the gene in a leukemia cell line with del(5q) inhibited its clonogenic properties [23]. Since then, JMJD1B mutations have been identified in Wilms tumor (a childhood kidney tumor), hepatoblastoma, acute myeloid leukemia, and Hodgkin lymphoma [36]. However, it is important to note that JMJD1B has both tumor-suppressive and tumor-promoting activities, which can vary depending on the specific tumor type. Therefore, the function of JMJD1B is likely cell type-dependent, and its effects are reflected in downstream targets. For example, the high expression of JMJD1B in breast cancer is associated with a better prognosis [37]; acute myeloid leukemia patients often exhibit low JMJD1B expression, and its overexpression can repress colony formation [23]. The depletion of JMJD1B increases cell proliferation and migration in certain human cell lines, as JMJD1B acts as a repressor of ANGPT1, a key regulator of angiogenesis [34]. On the other hand, JMJD1B can stimulate the expression of leukemogenic oncogenes by demethylating H3K9 at its promoters, suggesting a role in promoting oncogenesis [21]. The depletion of JMJD1A and JMJD1B in human colorectal cancer stem cells inhibits tumorigenic cell growth by promoting Wnt target gene transcription [35]. Furthermore, JMJD1B is upregulated in human hepatocarcinoma, and its knockout reduces cell proliferation by delaying the cell cycle [33]. Interestingly, a recent study identified JMJD1B as one of the six antioxidant genes in a prognostic gene signature for kidney renal clear cell carcinoma (KIRC) based on antioxidant genes [38]. Building on our studies, we propose an additional function of JMJD1B in cancer progression beyond gene expression, specifically in the regulation of the histone supply to the nucleus. Through a yet-unknown mechanism, the absence of JMJD1B leads to a defect in the proteosomal-mediated degradation of the histone chaperone tNASP, leading to an increase in its protein levels, resulting in the accumulation of newly synthesized histones H3/H4 in the cytoplasm, thereby blocking the histone supply to the nucleus (Figure 5). Our findings demonstrate that KDM3B/JMJD1B inhibits tumorigenic properties and genome instability in a murine melanoma model and correlates with oncogenic driver mutations, including p53, in human melanoma tumors. Taken together, our results underscore the pivotal role of the JMJD1B protein in maintaining genome stability, potentially acting as a safeguard against oncogenic mutations. This further highlights the potential role of JMJD1B in cancer progression beyond its established function in gene expression regulation.

## 4. Material and Methods

### 4.1. Generation of the JMJD1B Knockout in B16F10 Cells

To generate the knockout of the JMJD1B gene in the B16F10 cells, we utilized the http://crispr.mit.edu (accessed on 8 March 2019) design tool to select an optimal gRNA sequence targeting the first exon of the mouse JMJD1B gene. The selected gRNA sequence (5′-CAACAGCCGCTTGCCCACCG-3′) was cloned into the SpCas9-2A-GFP vector (Addgene PX458). The B16F10 cells were seeded into 6-well plates and then transfected with 1 µg of the JMJD1B gRNA-containing SpCas9-2A-GFP vector using Lipofectamine 2000 (Life Technologies, Carlsbad, CA, USA). Three days after the transfection, the GFP-positive cells were isolated through cell sorting, and 30.000 cells were plated on a 15 cm dish to allow them to grow as single colonies. A total of twenty-seven individual B16F10 clones were analyzed by Western blotting using the total cell extracts to detect the presence of JMJD1B. From these clones, we selected three (clones #16, #18, and #19), based on their growth characteristics, phenotypic traits, and lack of JMJD1B expression, as determined by the Western blot analyses, for further investigation.

### 4.2. Cell Culture

The B16F10 cells were cultured in Dulbecco’s Modified Eagle’s Medium (DMEM) supplemented with 2 mM of L-Glutamine, 100 units/mL of penicillin, 100 µg/mL of streptomycin, non-essential amino acids, 1 mM of sodium pyruvate, and 10% fetal bovine serum. The cells were grown at 37 °C with 5% CO_2_.

### 4.3. Cellular Extracts

The cells were seeded on 10 cm plates and allowed to reach an 80% confluency. Subsequently, they were trypsinized and washed with PBS. For the total cell extraction, the cells were resuspended in the RIPA buffer (20 mM of Tris HCl at a pH of 8.0, 150 mM of NaCl, 1.0% NP-40, 1.0% Sodium Deoxycholate, 1.0 mM of EDTA, and 0.1% SDS), supplemented with protease inhibitors, and incubated for 1 h at 4 °C. The cell suspension was then centrifuged at 14,000 rpm for 1 h at 4 °C. For the cytosolic and nuclear extraction, the cells were lysed according to the protocol described by [39]. Briefly, the cells were resuspended in a hypotonic buffer (10 mM of Tris at a pH of 7.9; 1.5 mM of MgCl_2_; 10 mM of KCl; 0.5 mM of DTT; and 0.2 mM of PMSF), supplemented with protease inhibitors, and lysed for 10 strokes with a type B Dounce homogenizer. The homogenates were centrifuged at 16,000 rpm for 1 h at 4 °C, and the supernatant was collected as the cytosolic extract (S100). The pellet was resuspended in a buffer containing 20 mM of Tris at a pH of 7.9, 1.5 mM of MgCl_2_, 0.42 M of NaCl, 0.2 mM of EDTA, 0.5 mM of DTT, 0.5 mM of PMSF, and 25% glycerol, and then incubated for 30 min at 4 °C. The homogenates were centrifuged again at 15,000 rpm for 30 min at 4 °C, and the supernatant was collected as the nuclear (NE) extract. The protein quantification was performed using a Bradford protein assay, and the samples were stored at −80 °C.

### 4.4. Antibodies

The following antibodies were used in this study: GAPDH (Santa Cruz Biotech, Dallas, TX, USA, sc-365062), β-actin (Sigma-Aldrich, Saint Louis, MI, USA, A5316), γH2AX (Abcam, Cambridge, UK, ab2893), H3 (Abcam, ab1791), H4 (Abcam, ab10158), HIRA (Abcam, ab20655), HSC70 (Abcam, ab19136), HSP90 (Abcam, ab13492), JMJD1B (Cell Signaling Technology, Danvers, MA, USA, 2621S), NASP (Abcam, ab181169), Topo I (Santa Cruz, sc-32736), anti-Mouse IgG HRP (Rockland, Philadelphia, PA, USA, 610-1302), anti-Rabbit IgG HRP (Rockland, 611-1322), and anti-Rat IgG HRP (Rockland, 612-1302).

### 4.5. Proliferation Assay

One thousand B16F10 cells from each line (WT; and JMJD1B KOs #16, #18, and #19) were seeded in 96-well plates containing the complete DMEM medium. After 24, 48, 72, and 96 h, the cells were washed one time with PBS, and a fresh DMEM medium was added containing 10 µL of CCK-8 (Cell Counting Kit-8, Sigma-Aldrich). After a two-hour incubation at 37 °C, the absorbance was measured at 450 nm. The doubling time of each cell was calculated using GraphPad Prism with an exponential non-linear fit. The statistical analyses were performed using a two-way ANOVA.

### 4.6. Soft Agar

The procedure was conducted following the protocol outlined by [40]. Initially, a bottom layer consisting of 1.5 mL of a 1:1 mixture of 1% agar and 2× complete DMEM medium was added to each well of a 6-well plate. Once the bottom layer solidified, a top layer consisting of 1.5 mL of a 1:1 mixture of 0.6% agar and 250 B16F10 cells from each line (WT; and JMJD1B KOs #16, #18, and #19) in a 1× complete DMEM medium was added on top. After 17 days of incubation, the colonies were manually counted and analyzed using a paired *t*-test.

### 4.7. Wound Healing Assay

The assay was conducted following the procedure described by [41]. Briefly, 5 × 10^5^ cells from the wild type and the JMJD1B knockout clones (#16, #18, and #19) were seeded in individual wells of 6-well plates. A diametrical line was drawn in each well as a reference. Once the cells reached an 80% confluency, they were washed with PBS, and the medium was replaced with a serum-free DMEM. The following morning, mitomycin C was added to a concentration of 8 µg/mL and incubated for 1 h. Wounds were created by marking three parallel diametric lines perpendicular to the reference line using a sterile p200 tip. The cells were then washed three times with PBS, and a medium containing 1% fetal bovine serum was added. Photographs of the wounds were captured at 0 and 8 h. The images were analyzed using ImageJ Version 1.54, and the data were plotted and statistically analyzed using the Student’s *t*-test with GraphPad Prism 9.

### 4.8. Tumor Growth

In total, 1 × 10^6^ B16F10 melanoma cells (WT; and JMJD1B knockouts #18 and #19) were intradermally injected into the RAG KO C57BL/6 mice. The tumor growth was monitored by measuring the perpendicular diameters of the tumor with calipers every other day. The tumor volume was calculated using the following formula: V = (D × d^2^)/2, where V represents the volume (mm^3^), D represents the larger diameter (mm), and d represents the smaller diameter (mm). The mice were euthanized when the mean tumor diameter reached 15 mm or according to the approved ethical protocol.

### 4.9. Microccocal Nuclease (MNase) Digestion

The B16F10 cells of each line (WT; and JMJD1B knockouts #16, #18, and #19) were seeded on 15 cm plates. Once the cells reached confluency, they were trypsinized, washed with PBS, and resuspended in 50 µL of MNase Buffer (0.25 M of sucrose; 15 mM of NaCl; 60 mM of KCl; 10 mM of Tris at a pH of 7.5; 5 mM of MgCl_2_; 1 mM of CaCl_2_; 0.5% Triton X-100; 0.1 mM of PMSF; and 0.5 mM of DTT). Two µL of MNase (40 U/mL) were added to the cell suspension, and the cells were incubated at 37 °C. At time points 0, 7, 10, 15, and 20 min, 20 µL aliquots were taken and transferred to 1.5 mL tubes containing 2 µL of 0.1 M EDTA, and then kept at 4 °C. After the Proteinase K treatment, the DNA samples were resolved on a 1.5% agarose gel, stained with GelRed, and photographed. The DNA band sizes were compared to a 100 bp DNA ladder (Invitrogen, Waltham, MA, USA). The images were analyzed using ImageJ Version 1.54 software, and the resulting peaks were graphed using GraphPad Prism 6.

### 4.10. Comet Assay

The comet assay was performed following the protocol described by [42]. Briefly, the B16F10 cells from each line (WT; and JMJD1B knockouts #16, #18, and #19) were seeded on 10 cm plates. Once the cells reached confluency, they were trypsinized, washed with PBS, and suspended in 1% low melting agarose. Approximately 16,000 cells were diluted in 1 mL of 1% low melting agarose and transferred onto microscope slides covered with 1% agarose. The slides were then placed in a Coplin jar containing a cold lysis buffer (2.5 M of NaCl; 100 mM of EDTA; 10 mM of Tris-Base at a pH of 10.5; 1% DMSO; and 1% Triton x-100) and kept overnight at 4 °C, protected from light. The next day, the slides were incubated for 30 min at 4 °C, protected from light, in an electrophoresis buffer (300 mM of NaOH; 1 mM of EDTA; and 1% DMSO at a pH of 13). The electrophoresis was performed at 1 V/cm^2^ for 1 h at 4 °C, protected from light. After the electrophoresis, the slides were transferred back to the Coplin jar, washed three times with cold distilled water, stained with GelRed Nucleic Acid Gel Stain, and air-dried overnight, protected from light. On the following day, the slides were imaged using a fluorescence microscope. The comets were measured using the OpenComet plugin in ImageJ, and the data were analyzed using GraphPad Prism 6.

### 4.11. Transcriptomic Analyses of Human Melanoma Tumors

The records of patients with cutaneous melanoma (SKCM) from the Cancer Genome Atlas (TCGA) project were accessed through the Genomic Data Commons Data Portal (GDC) repository of the National Cancer Institute. We obtained the RNAseq-derived normalized gene expression as transcripts per million (tpm) and transformed it to the z-score. The occurrence of single nucleotide variant (SNV) mutations was assessed for all the sequenced genes. The mutational load was defined by the total mutation count with a log2(n + 1) transformation. The association between the mutational load and JMJD1B/KDM3B expression was evaluated by the Spearman’s rank correlation coefficient, along with the correlation between the expression of different genes. The association between JMJD1B/KDM3B expression and the presence of SNV mutations was determined by a univariate logistic regression. A significance level of α = 0.05 was considered significant.

### 4.12. Ethics Statement

All the studies were carried out according to the 8th edition of the Guide for the Care and Use of Laboratory Animals. The experimental protocols were approved by the IACUC at the Fundacion Ciencia & Vida, including those involving anesthesia, pain, distress, and euthanasia (Nº P058/2023). The ethical approval date was 20 September 2023.

## 5. Conclusions

Our findings underscore the significance of JMJD1B/KDM3B in preserving genome integrity through the assurance of the histone supply to the nucleus, extending its role beyond the regulation of gene expression. JMJD1B stands out as a key contributor to maintaining genome stability in the context of melanoma, potentially serving as a protective barrier against the occurrence of oncogenic mutations.

## Data Availability

The datasets used and/or analyzed during this current study are available from the corresponding author on reasonable request.

## Supplementary Materials

The following supporting information can be downloaded at https://www.mdpi.com/article/10.3390/ijms251910689/s1.
