# Supplementary material for: Unraveling the Role of JMJD1B in Genome Stability and the Malignancy of Melanomas
Source: Int J Mol Sci. 2024 Oct 4;25(19):10689. doi: 10.3390/ijms251910689 (PMC11476393; doi:10.3390/ijms251910689)
Supplement: Supplementary file 1 [file ijms-25-10689-s001.zip › ijms-3216203-supplementary materials.pdf]

SUPPLEMENTARY FIGURE

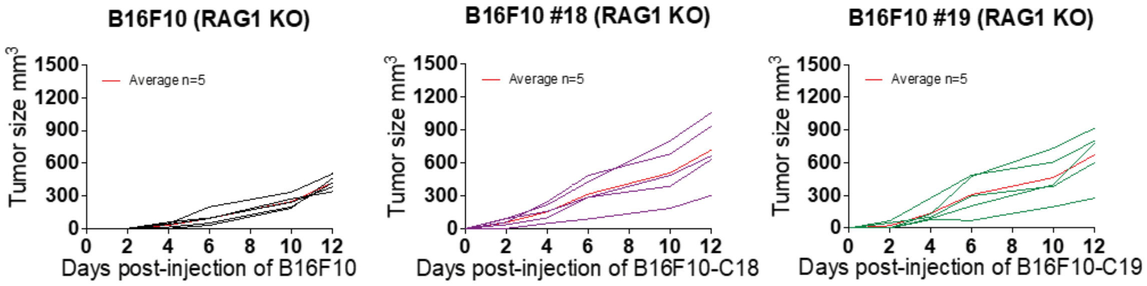

Supplementary Figure S1. In vivo tumor growth. RAG1 KO C5BL/6 mice were intradermally injected with $1x10^6$ wild-type (WT) and JMJD1B knockout clones #18, and #19 B16F10 melanoma cell lines. Tumor growth was evaluated in the subsequent days. The graph presents individual tumor growth curves of all conditions; B16F10-WT (black lines), B16F10 #18 (purple lines) and B16F10 #19 (green lines), red lines show the average of each group.
